# Somatic *SDHA* mutations in paragangliomas in siblings: Case report of 2 cases

**DOI:** 10.1097/MD.0000000000022497

**Published:** 2020-10-09

**Authors:** Yen-Chun Huang, Hsiao-Huang Chang, Ming-Huang Chen, Kuo-Hung Huang, Anna Fen-Yau Li, Chien-Hsing Lin, Yi-Ming Shyr, Wen-Liang Fang

**Affiliations:** a Division of General Surgery, Department of Surgery, Taipei Veterans General Hospital; b School of Medicine, National Yang-Ming University; c Division of Cardiovascular Surgery, Department of Surgery; d Department of Oncology, Center of Immuno-Oncology; e Department of Pathology, Taipei Veterans General Hospital; f Genome Research Center, National Yang-Ming University, Taipei City, Taiwan.

**Keywords:** paraganglioma, sibling, somatic *SDHA* mutation

## Abstract

**Rationale:**

Paragangliomas (PGLs) are rare neuroendocrine tumors that are strongly influenced by genetics, and succinate dehydrogenase-deficient PGLs appear to constitute one of the most important categories. Interestingly, somatic PGLs only possess genomic alterations involving the *SDHB* and *SDHD* subunits, and no *SDHA* alterations have been described. Here, we are presenting the clinical and genetic analyses of 2 cases with the first somatic *SDHA* variant identified in PGLs.

**Patient concerns:**

Here, we reported 2 family members with the diagnosis of PGL. Patient 1 is a 55-year-old woman with a functionally perigastric PGL that co-occurred with a gastric gastrointestinal stromal tumor (GIST), and patient 2 is a 43-year-old woman with a nonfunctionally pericardial PGL, who was the younger sister of the first patient.

**Diagnoses:**

Imaging surveys of the 2 cases depicted the presence of a perigastric and a pericardial mass, respectively. A diagnosis of paragangliomas was established by immunohistochemistry (IHC).

**Interventions:**

Both patients underwent single-stage resection of the lesion after preoperative oral α-adrenoceptor therapy for 2 weeks. We later performed comprehensive genomic profiling on the tumor samples, including PGL and GIST from patient 1 and PGL from patient 2, and searched for novel actionable mutations, including in all succinate dehydrogenase subunits, as the IHC results were negative for *SDHB*.

**Outcomes:**

Both patients had an uneventful recovery after surgery and the sequencing showed a novel somatic variant in the *SDHA* gene on chromosome 5q11 (c.1945_1946delTT). Regular follow-up with biochemical testing and image studies showed no evidence of recurrence after a year for patient 1 and 6 years for patient 2.

**Lessons:**

PGLs often lead to considerable diagnostic difficulty due to their multiple anatomical locations and variable symptoms, as presented by our cases. The comprehensive use of images and plasma/urine catecholamine measurement can aid the diagnosis of PGLs. In addition, our findings also demonstrate the usefulness and importance of genetic analysis of *SDHA* mutations in patients exhibiting *SDHB* IHC-negative PGL. Additional studies utilizing comprehensive genomic profiling are needed to identify the group of PGLs harboring this *SDHA* genomic alteration.

## Abstract 2

Supplemental Digital Content is available in the text

## 1 Introduction

Paragangliomas (PGLs) are rare neuroendocrine tumors originating from the neural crest–derived chromaffin cells, which arise from either the sympathetic or parasympathetic paraganglia.^[1]^ The annual incidence of PGLs is approximately 1/100,000, with a peak in the fourth decade of life; women have a higher incidence rate than men among patients with sporadic tumors (71% vs 29%), whereas the hereditary type does not predominantly occur in 1 sex.^[2,3]^ Usually, sympathetic PGLs derived from sympathetic tissue in the abdominal or thoracic locations and parasympathetic PGLs develop mostly in the head and neck region.

Studies on the genetic aspects of PGLs have identified several susceptibility genes as a direct cause of or a prognostic factor for PGLs. Sporadic mutations, which have been reported in only Rearranged during transfection, von Hippel-Lindau, Neurofibromatosis type I, MYC-associated factor X, hypoxia-inducible factor 2A, and recently *SDHB* and *SDHD*, explain the remaining 60% of cases, and more than one-third of those cases are associated with a somatic alteration in a predisposing gene.^[4]^ The *SDHx* group of genes, which are tumor-suppressor genes, encode the subunits of succinate dehydrogenase (SDH) (*SDHA*, *SDHB*, *SDHC*, and *SDHD*). These subunits assemble to assist in oxidizing succinate to fumarate in the Krebs cycle and in electron transport to the ubiquinone pool. Germline mutations in these 5 tumor suppressor genes (TSGs) have been identified in patients with hereditary PGL^[5–9]^ and more recently, have been reported in gastrointestinal stromal tumors (GISTs), renal cell carcinoma, and pituitary adenomas.

To date, except for somatic *SDHB* and *SDHD* mutations, each of which has been reported only once,^[10,11]^ only germline mutations have been described in all SDH subunit genes, even among mutations in apparently sporadic PGLs. Here, we described the clinicopathologic features, genetic mutations, and prognoses of 2 family members; the younger sister had a cardiac PGL and a GIST, whereas the older sister had a PGL near the esophagogastric junction. Both had the same *SDHA* mutation (c.1945_1946delTT) in the tumor tissue; therefore, they are the first reported patients to have PGLs with somatic *SDHA* genomic alteration.

## 2 Case presentation

### 2.1 Patient 1

A 55-year-old woman, who was the elder of the 2 sisters, presented at our hospital because of a headache and uncontrolled hypertension. Three months before the current admission, she experienced a few episodes of a pulsatile headache that was associated with palpitations, heat intolerance, and diaphoresis. The patient denied nausea/vomiting, chest pain, shortness of breath, abdominal pain, or bowel or urinary symptoms. On physical examination, the patient was awake and oriented; the headache intermittently occurred. Her pulse was 92 beats per minute, her blood pressure (BP) was 230/120 mm Hg, and her respiratory rate was 16 breaths per minute. Mild flushing of the face was noted and the results of the remaining physical examinations were normal. Eventually, a total of 4 types of antihypertensive drugs were then administered with repeated evaluations and drug adjustments. However, her BP was persistently uncontrolled, with an average of 180/90 mm Hg; the headache and accompanying symptoms, including the palpitations and diaphoresis, subsided with time. The hematological and biochemical workup data were within the normal limits, so as the results of serum tumor markers and 24-hour urine catecholamine and vanillylmandelic acid tests (Table 1).

**Table 1:**
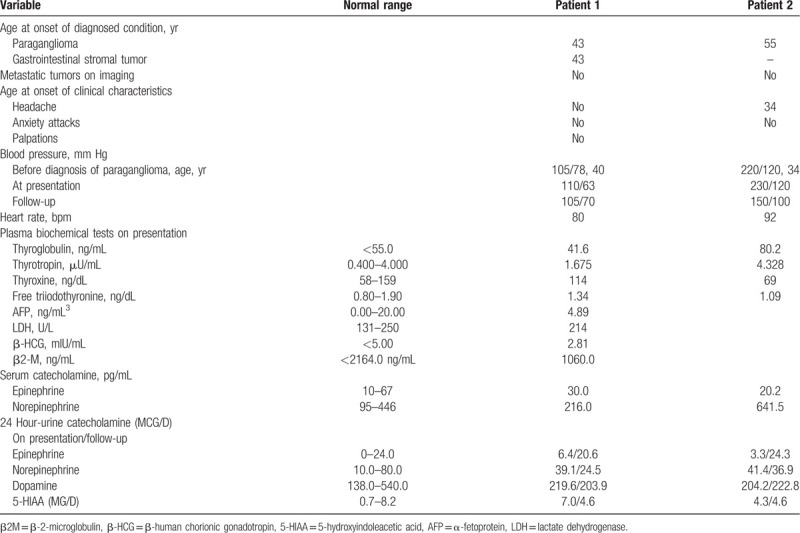
Age at presentation, clinical characteristics, and laboratory values of the patients.

**Figure 1:**
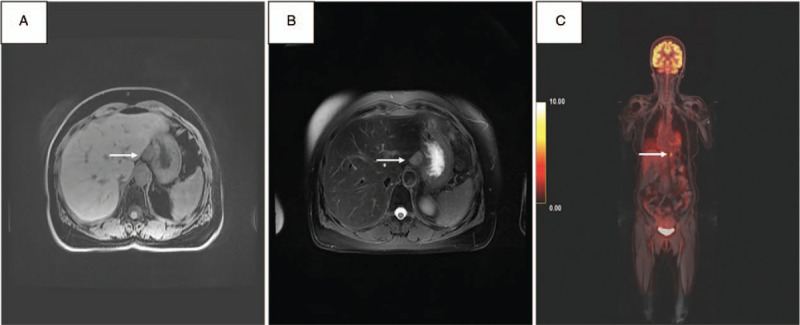
A–C, An axial PET-CT/MRI hybrid scan of the abdomen showed a well-circumscribed, homogenous mass in the gastrohepatic ligament. It was slightly (A) hyperintense on T1 imaging, (B) hyperintense on T2 imaging. C, An increased 18F-fluorodeoxyglucose uptake (SUVmax 7.4) can also be noted in the gastrohepatic ligament on PET-CT scan.

**Figure 2:**
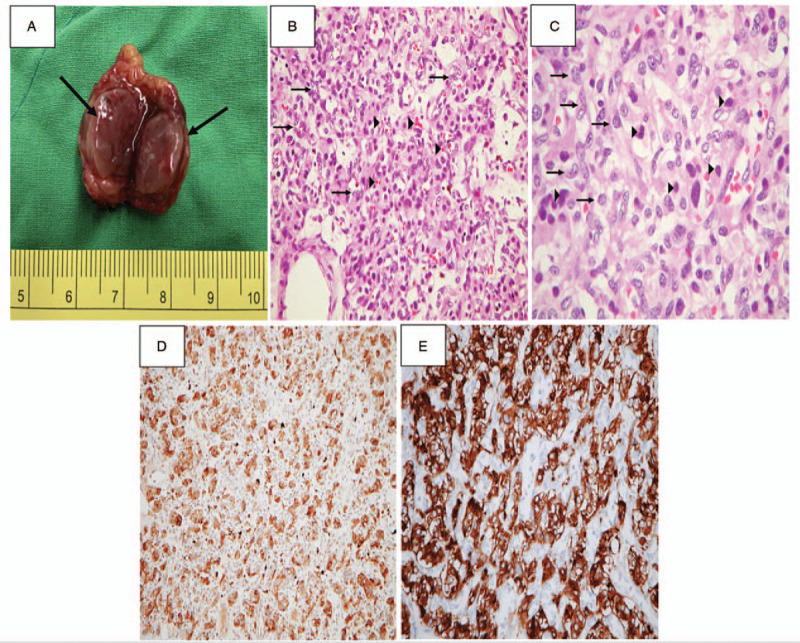
A–E, Resection specimens (patient 1). A, A photograph of the resection specimen shows a tannish red, fleshy mass (arrows) within the lesser omental tissue. B, Hematoxylin and eosin micrograph shows a mass composed of well-defined nests of tumor cells (arrows) bound by a delicate fibrovascular stroma (arrowhead). C, At higher magnification, neoplastic cells with fine-to-coarse puncta of chromatin (arrows) are surrounded by eosinophilic cytoplasm. D, E, Immunohistochemical staining for chromogranin A and synaptophysin is strongly positive in tumor cells, respectively.

Sonograms of the abdomen showed an ovoid-shaped hypoechoic nodule approximately 1.8 × 1.4 cm in size close to the right side of the esophagogastric junction, and no other significant findings were noted. Further evaluation with 18F-fluorodeoxyglucose whole-body Positron Emission Tomography/Magnetic resonance (PET-CT/MR) hybrid scanning of the abdomen showed a well-circumscribed, homogenous mass in the gastrohepatic ligament adjacent to the body of the stomach and immediately above the pylorus, below the left lobe of liver, and anterior to the abdominal aorta. It was 2.3 × 1.7 mm in size, slightly hyperintense on T1 imaging (Fig. 1A), and hyperintense on T2 imaging (Fig. 1B). In addition, an area of increased uptake corresponding to the nodule observed on the abdominal sonogram was depicted, and no other FDG-avid lesions were detected elsewhere (Fig. 1C). The patient later underwent robotic tumor excision after preoperative oral α-adrenoceptor therapy for 2 weeks. The pathological results showed grossly tannish red, fleshy, and encapsulated within soft, yellow, and fatty lesser omental tissue. The interface between the mass and the omental fat was regular and clear, which was a macroscopic finding suggestive of noninfiltrative growth (Fig. 2A). H&E micrographs (Fig. 2B) showed a highly cellular mass composed of well-defined nests of tumor cells bound by a delicate fibrovascular stroma. At higher magnification (Fig. 2C), the neoplastic cells had round, regular central nuclei with fine-to-coarse puncta of chromatin, which is a finding typical of neuroendocrine differentiation. Mitotic figures were rarely observed. Immunohistochemistry (IHC) staining for chromogranin A and synaptophysin, both of which are neuroendocrine markers (Fig. 2D and E), was diffusely and strongly positive in the tumor cells. The pathological findings concluded the diagnosis of PGL.

### 2.2 Patient 2

**Figure 3:**
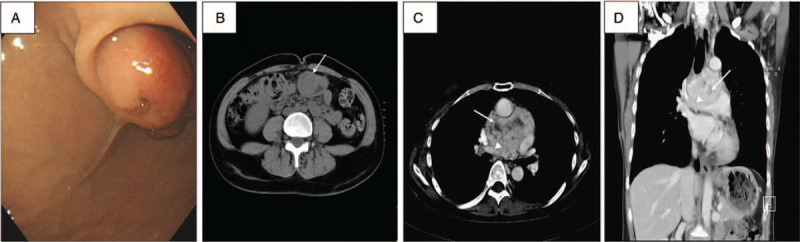
A–D, Esophagogastroduodenoscopy and computed tomography (CT) scan of the chest. A, Esophagogastroduodenoscopy showed a submucosal mass measuring 4 × 2.5 × 2.5 cm in size, with focally ulcerated mucosa. B, A CT scan of the abdomen depicted a 5 × 3.5 cm well-defined hypervascular tumor at the lower gastric body. C, D, Axial and sagittal images of the chest depict a 7.4 × 6.6 cm hypervascular and heterogeneous mass in the precarinal region (arrows) with the encasement of Right pulmonary artery (arrowheads).

**Figure 4:**
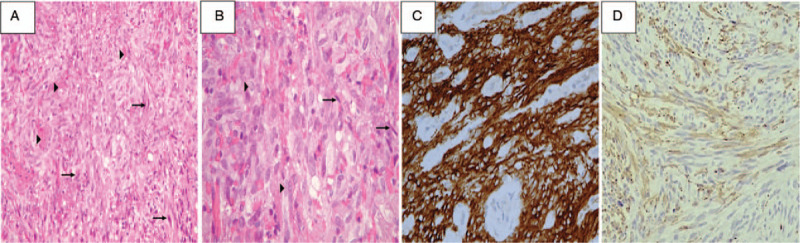
A–D, Resection specimens of gastrointestinal stromal tumor (GIST; patient 1). A, Hematoxylin and eosin (H&E) staining of a section of the gastric GIST shows a mass rich in vascularity (arrowhead) and spindle-shaped neoplastic cells with inconspicuous nucleoli (arrows) infiltrating in the submucosa. B, At higher magnification, neoplastic cells (arrows) are bland-appearing, with eosinophilic cytoplasm. C, D, Immunohistochemistry (IHC) staining for CD117 and desmin was strongly positive for CD117 but negative for desmin, respectively.

A 43-year-old woman, who was the younger of the 2 sisters reported here, was admitted to our hospital because of an incidentally discovered mediastinal mass. The patient had been in her usual good health until a year before this evaluation, when a gastric submucosal tumor was identified on esophagogastroduodenoscopy (Fig. 3A) after the occurrence of melena passage and severe anemia. Preoperative endoscopic biopsy was simultaneously performed and suggested the presence of GIST. Computed tomography (CT) scan of the abdomen and pelvis depicted a 5 × 3.5 cm hypervascular mass at the lower gastric body (Fig. 3B) and revealed no signs of metastasis. Subtotal gastrectomy with Billroth-II reconstruction was performed. On pathological examination, H&E staining of a section of the gastric GIST (Fig. 4A) showed a highly cellular mass rich in vascularity with spindle-shaped neoplastic cells with small or inconspicuous nucleoli infiltrating the submucosa. At higher magnification (Fig. 4B), the neoplastic cells were bland-appearing with uniform and syncytial-appearing eosinophilic cytoplasm. Mitotic figures were rarely observed. IHC staining for CD117 and desmin were strongly positive for CD117 but negative for desmin (Fig. 4C and D). The pathological reports confirmed the diagnosis of a GIST.

**Figure 5:**
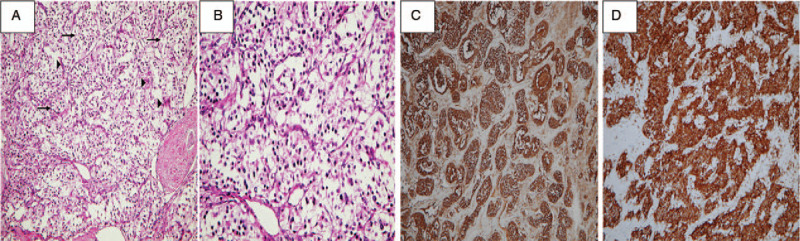
A–D, Resection specimens of paraganglioma (PGL) (patient 1). A, H&E staining of a section of the PGL shows classic zellballen pattern. B, At higher magnification, neoplastic cells (arrows) have round nuclei with low mitotic activity. C, D, Immunohistochemical staining for chromogranin A and synaptophysin is strongly positive in tumor cells, respectively.

Approximately 7 weeks later, during regular outpatient department follow-up, a mass was incidentally identified in the precarinal space on chest CT scan. She denied any accompanying symptoms, such as fever, weight loss, upper respiratory symptoms, nausea, vomiting, chest pain, or dyspnea. Her BP was 110/63 mm Hg and her pulse rate was 80 beats per minute. The hematological examination and biochemical tests including the thyroid panel and tumor marker profile (Table 1) were all within normal limits. An electrocardiogram revealed the presence of sinus rhythm and chest radiography showed widening of the upper mediastinum with mild bilateral apical pleural thickening, suspecting enlarged lymph nodes or the involvement of other soft tissue. A CT scan of the chest confirmed the presence of a 7.4 × 6.6 cm hypervascular mass with necrosis between the ascending aorta and the dome of the left atrium with encasement of the right pulmonary artery (Fig. 3C and D). Diagnostic core biopsy of the mediastinal mass confirmed the diagnosis of PGL composed of uniform cells with round nuclei that grew in nests surrounded by sustentacular cells. Consequently, the level of serum catecholamine was checked, and the result was within normal limits (Table 1). Open surgical excision was performed via a median sternotomy in 2013. On pathological surveys, H&E staining of a section of the PGL (Fig. 5A) showed nest-like clusters of uniform, round-to-polygonal chief cells surrounded by delicate, richly vascular tissue and sustentacular cells, referred to as the zellballen pattern. At higher magnification (Fig. 5B), the neoplastic cells had round, regular central nuclei with low mitotic activity and small to moderate amounts of eosinophilic cytoplasm. IHC staining for chromogranin A and synaptophysin (Fig. 5C and D) was also strongly positive in the tumor cells. The pathological findings confirmed the diagnosis of PGL.

### 2.3 SDH immunohistochemical staining and genetic analysis

**Figure 6:**
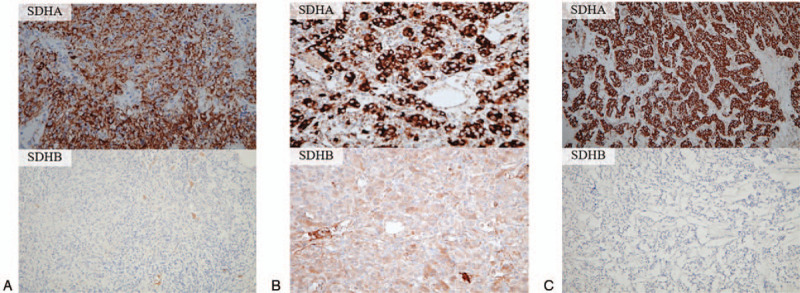
A–C, Immunohistochemical staining. Positive staining for *SDHA* but decreased staining for *SDHB* are demonstrated in (A) gastric gastrointestinal stromal tumor (GIST) in patient 1, (B) pericardial paraganglioma (PGL) in patient 1, (C) gastric PGL in patient 2.

**Figure 7:**
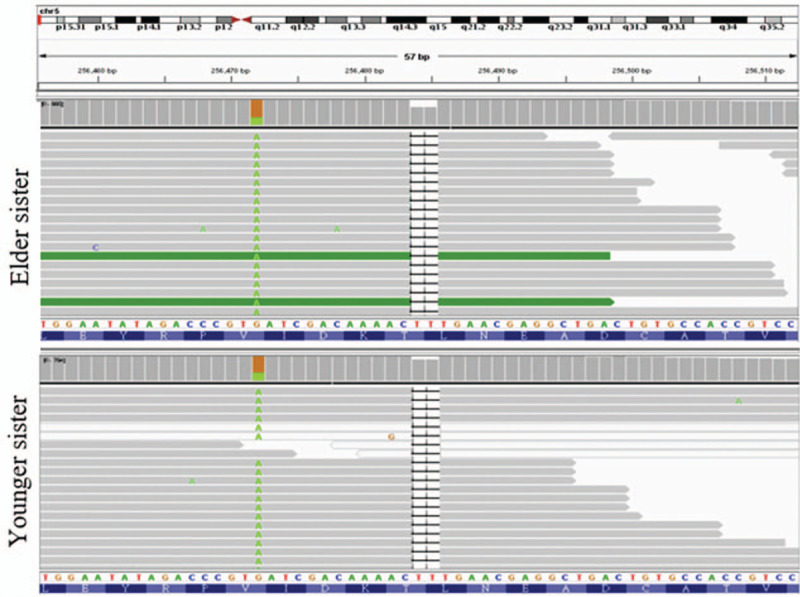
Identification of the *SDHA* mutation. The panel shows the results of genotype sequencing of *SDHA* in affected members of the proband's family. An enlarged view of the analyzed cosegregating region on chromosome 5 is shown above, with nucleotide positions shown below. Double-stranded nucleotide sequences are shown in the bottom of the chromatograms, with the corresponding amino acids labeled with the 1-letter symbols below. We identify a frameshift-creating deletion of two thymines at nucleotide position 1945 (c.1945_1946delTT) that results in premature stop at codon 653 (p.Leu649GlufsTer4).

To evaluate the protein expression levels of *SDHA* and *SDHB*, IHC analyses (Supplementary Appendix, http://links.lww.com/MD/E920) were performed on the tumor tissues of both patient 1 and patient 2. The staining results showed loss or decreased *SDHB* expression but preserved *SDHA* expression in the examined neoplasms, as compared with adjacent normal tumor tissues (Fig. 6A–C). We further analyzed SDH subunit genes via next-generation sequencing (Supplementary Appendix, http://links.lww.com/MD/E920). A novel variant in *SDHA* on chromosome 5p15 was identified in the tumor tissues of both patients but not in the peripheral blood, indicating that it was a somatic mutation. This *SDHA* variant results in an elimination of 2 thymines at base 1945 (c.1945_1946delTT), leading to a frameshift mutation. The frameshift was predicted in codon 649 and causes in a premature stop at codon 653 (p.Leu649GlufsTer4). The alternate variant frequencies were calculated to be 29.47% for patient 1 and 15.58% for patient 2; no mutations were discovered in the other SDH subunits (Fig. 7).

Both patients had an uneventful recovery after surgery. Regular follow-up with biochemical testing (Table 1) and image studies showed no evidence of recurrence after a year for the elder sister and 6 years for the younger sister.

## 3 Discussion

PGLs have been called the ”great mimickers” by physicians owing to their variable symptoms and locations.^[12]^ The clinical course of the elder sister (patient 2) highlights the diagnostic challenge posed by the diagnosis of tumors near the esophagogastric junction. The patient was mostly asymptomatic with her only symptoms being an intermittent pulsatile headache and diaphoresis, which even subsided approximately a month before diagnosis. In retrospect, newly abrupt onset of hypertension at the relatively young age of 34 with previously normal BP and drug-resistant hypertension were both significant clues to the presence of a PGL. In scenarios like our cases, studies have recommended that biochemical testing is needed if CT attenuation of the incidentally discovered mass is >10 Hounsfield units and suggested clearly elevated values of fractionated catecholamines (>2 times the upper limit of the normal range) to be diagnostic.^[13–15]^ Nonetheless, the subsequent biochemical surveys were all within normal limits, and only a relatively high level of serum norepinephrine (641.5 pg/mL) was noted. Here, we indeed could not exclude the possibility of essential hypertension with a nonfunctioning PGL within our case. However, paroxysmal hypertension, a consequence of episodic secretion of catecholamines by functioning PGL, has been reported earlier, which was consistent with the clinical course of our case. If so, our hypothesis of the normal catecholamines level is that we measured them at the time that the PGL does not secrete; hence, we believed that repeated testing should have been administered.

On radiological examinations, the rapid perfusion on first-pass MRI showed a tumor rich in vascularity. An initial diagnosis of a GIST was suspected considering the patient's age, the location of the tumor, the finding on imaging of a smoothly contoured solid mass, and fact that GISTs are the most common nonepithelial neoplasms in the gastrointestinal tract. There are currently no specific criteria to accurately distinguish GISTs from PGLs, although relatively greater degrees of hypervascularity have been noted in PGLs than in GISTs.^[16,17]^ In addition, preoperative biopsy is not recommended for resectable lesions due to the possibility of complications, including a catecholamine crisis, severe bleeding, or subsequent fibrosis at the operative site that might affect the possibility of definitive surgery.^[18,19]^ Consequently, it is easy to misdiagnose PGL, especially when it occurs in a rare location near the gastrointestinal tract, as in this case, further making tissue-based confirmation difficult. To the best of our knowledge, only 11 cases of perigastric PGLs have been reported in the literature.^[20–28]^ Regarding the younger sister (patient 1), who had a previous personal history of GIST and was similarly asymptomatic, metastasis from GIST was initially considered, and the difficulties achieving the accurate diagnosis would have persisted if a preoperative biopsy had not been taken. Therefore, genetic testing and surveillance for the diagnosis of hereditary PGL are practical and necessary, according to the recommendation of a recently reported large PGL series.^[29,30]^

SDH genes function as TSGs and mutations in the components of the SDH enzyme are considered to be the most prevalent of all the recognized hereditary genetic abnormalities causing PGL, accounting for up to 50%.^[31,32]^ Notably, previous research has identified large numbers of mutations in the *SDHB* and *SDHD* genes in PGL, but the *SDHA* gene has been a relatively minor focus. This could be explained by the markedly lower disease penetrance rate and frequency of the loss of 5p15, which is the chromosomal region containing the *SDHA* locus.^[33–36]^ Moreover, PGLs associated with heterozygous *SDHA* mutations have been identified in only 95 cases in the literature databases, with 32 different loss-of-function or missense *SDHA* variants being observed.^[34]^ However, no genetic link between a somatic *SDHA* variant and PGL has ever been recorded, even though 2 previous reports have identified specific somatic mutations of the *SDHB* gene (c.299C > T, p.S100F) and *SDHD* gene (c.242C > T, p. P81L) that contribute to PGL.^[10,11]^

The rare variant (c.1945_1946delTT) identified in our patient is, to the best of our knowledge, the first somatic *SDHA* mutation associated with PGL. We have provided several lines for evidence to support our finding that this *SDHA* variant has a correlation between the development of true sporadic PGL or potentially relevant cancer. First, we found complete segregation of this variant within all the tested neoplasms of both affected patients. Second, we found loss of *SDHB* expression in tumor tissues of PGLs and GIST from variant carriers but not in normal tissue, suggesting the dysfunction of the SDH enzyme. These genetic results are consistent with an etiological connection between the immunohistochemical *SDHB*-negativity, in which this somatic mutation could lead to SDH protein instability like the other *SDHx* mutants. In addition, the relationship between this variant and PGL has not been recorded in the available genome databases, although 5 different samples, including melanoma, spinal cord astrocytoma, esophageal squamous cell carcinoma, and ovarian carcinoma, from 4 studies were documented as having the same somatic mutation variant.^[37–40]^

Interestingly, previous reports have shown that *SDHA* IHC of tumor tissues can efficiently detect the presence of *SDHA* germline mutations by exhibiting immunohistochemically negative staining.^[33,41]^ However, both of our patients had positive IHC staining for the SDHA protein. We hypothesized that the intratumor heterogeneity and haploinsufficiency could explain this contradictory result if the majority of the *SDHA* gene in the neoplasms was unmutated, thereby preserving the conformational structure of most of the SDHA protein. The alternate variant frequency was calculated to be 15.58% for patient 1 and 29.47% for patient 2, which could further support the validity of tumor heterogeneity as an explanation. In addition, studies have also shown that the growth of the entire tumor may be influenced by a minor tumor subpopulation with a survival advantage, thereby actively maintaining tumor heterogeneity.^[42,43]^ Moreover, haploinsufficiency might act synergistically with oncogenic signals in a tissue-specific manner under TSG-related tumorigenesis.^[44]^ Inconsistent immunohistochemical results were also observed for 3 discrepancies that occurred in *SDHA*, and likewise, showed *SDHB*-negative but *SDHA*-positive immunohistochemical staining (c.562C > T; p.Arg188Trp, c.818C > T; p.Thr273Ile, and c.1361C > A; p.Ala454Glu).^[45,46]^

## 4 Conclusions

The identification, characterization, diagnosis, and management of PGLs should be based on complete clinical, biochemical, radiologic, and morphological data due to their variable nonspecific signs and symptoms that overlap with a wide variety of other conditions. Our cases show the challenges faced when diagnosing PGL, including the possibility of misdiagnosing a PGL as a GIST due to the unique location, nearly asymptomatic behavior and potential co-occurrence. In addition, our study approved the usefulness and importance of genetic analysis of *SDHA* mutations in a family exhibiting *SDHB* IHC-negative PGL. It not only helps us ascertain the clear etiological role played by the *SDHA* alleles in PGL tumorigenesis but also assists us in clarifying the correlations between the genotype and phenotype of *SDHA* mutations, elucidating the risks to variant carriers and providing valuable information for targeted individual genetic counseling.

## Acknowledgments

The authors thank Dr Chien-Hsing Lin for the support with genetic analysis and Dr Anna Fen-Yau Li for the immunohistochemical stain. This research was supported by Taipei Veterans General Hospital, Taiwan (V109C-105). None of the sources of funding played a role in the study design, data collection, the analysis and interpretation of data, the writing of the manuscript, or the decision to submit the manuscript for publication.

## Author contributions

**Data curation:** Yen-Chun Huang, Chien-Hsing Lin.

**Investigation:** Wen-Liang Fang, Chien-Hsing Lin.

**Methodology:** Anna Fen-Yau Li. Chien-Hsing Lin.

**Manuscript writing:** Yen-Chun Huang.

**Manuscript revision and approval:** Wen-Liang Fang, Su-Shun Lo, Chew-Wun Wu, Yi-Ming Shyr.

## Abbreviations

Abbreviations: 5-HIAA = 5-hydroxyindoleacetic acid, BP = blood pressure, CT = computed tomography, GIST = gastrointestinal stromal tumor, IHC = immunohistochemistry, PGL = paraganglioma, SDH = succinate dehydrogenase, TSG = tumor suppressor gene.
